# Dietary Administration of Postbiotics from *Vibrio proteolyticus* DCF12.2 Enhanced Intestinal Integrity, Microbiota, and Immune Response in Juvenile Gilthead Seabream (*Sparus aurata*)

**DOI:** 10.3390/ani15131982

**Published:** 2025-07-05

**Authors:** Olivia Pérez-Gómez, Sonia Rohra-Benítez, Marta Domínguez-Maqueda, Isabel M. Cerezo, Alba Galafat, Eduardo Martínez-Manzanares, Juan Miguel Mancera, Francisco Javier Alarcón-López, Jorge García-Márquez, Miguel Ángel Moriñigo, Salvador Arijo

**Affiliations:** 1 Departamento de Microbiología, Facultad de Ciencias, Instituto Andaluz de Biotecnología y Desarrollo Azul (IBYDA), Universidad de Málaga, Ceimar-Universidad de Málaga, Campus Universitario de Teatinos s/n, 29071 Málaga, Spain; olipergom@uma.es (O.P.-G.); soniarb@uma.es (S.R.-B.); martadm@uma.es (M.D.-M.); emmanzanares@uma.es (E.M.-M.); morinigo@uma.es (M.Á.M.); 2 Departamento de Biología y Geología, Escuela Superior de Ingeniería, Universidad de Almería, Ceimar-Universidad de Almería, 04120 La Cañada de San Urbano, Spain; ico396@ual.es (I.M.C.); agd056@ual.es (A.G.); falarcon@ual.es (F.J.A.-L.); 3 Departamento de Biología, Facultad de Ciencias del Mar y Ambientales, Instituto Universitario de Investigación Marina (INMAR), Universidad de Cádiz, Ceimar-Universidad de Cádiz, 11510 Cádiz, Spain; juanmiguel.mancera@uca.es; 4 LifeBioencapsulation S.L., Parque Científico PITA, 04131 El Alquián, Spain

**Keywords:** aquaculture, aquafeeds, gut health, immune response, fish nutrition, postbiotic

## Abstract

The use of postbiotics for dietary fortification in aquaculture is gaining increasing attention due to their potential immunomodulatory and gut health benefits. In this study, we evaluated the effects of postbiotics derived from *Vibrio proteolyticus* DCF12.2 on intestinal histology, microbiota composition, and the expression of genes related to epithelial integrity and inflammation in juvenile gilthead seabream (*Sparus aurata*). Fish were fed either a control (CRTL) diet or the postbiotic-supplemented diet (VP) for 62 days. At the end of the feeding trial, a lipopolysaccharide (LPS) challenge was conducted to evaluate the immune response in fish. Histological analysis revealed a healthy mucosa in both groups, though fish fed the VP diet reduced fold height and mucosal layer thickness, alongside a significant increase in goblet cells. Microbiota profiling indicated higher alpha diversity and significant shifts in community composition in the VP group, including enrichment of potentially beneficial genera (*Pseudomonas, Sphingomonas*) and depletion of opportunistic taxa (*Enterococcus, Stenotrophomonas*). After the feeding trial, fish fed the VP diet exhibited downregulation of pro-inflammatory markers (*tnfα, cox2*). Following LPS challenge, *cdh1*—a key epithelial adhesion protein required for maintaining intestinal barrier integrity—expression was upregulated significantly in the VP group, suggesting enhanced epithelial resilience. These findings demonstrate that dietary fortification with *V. proteolyticus*-derived postbiotics supports mucosal health as well as modulates the intestinal microbiota and immune responses in gilthead seabream juveniles, offering a promising strategy for functional aquafeed development in sustainable aquaculture.

## Simple Summary

Maintaining fish health is essential for the growth of sustainable aquaculture. One emerging strategy is the use of postbiotics as functional feed additives. In this study, we evaluated the dietary inclusion of postbiotics derived from *Vibrio proteolyticus* in juvenile gilthead seabream. Fish were fed either a standard diet or a postbiotic-supplemented diet for 62 days. At the end of the feeding period, we examined their intestinal health, the composition of gut bacteria, and their immune response. Fish that received the postbiotic diet showed a healthy intestinal structure and changes in the bacterial community that favored beneficial microbes while reducing potentially harmful ones. Additionally, these fish had lower levels of inflammation-related gene activity, suggesting a more balanced immune status. After being exposed to a lipopolysaccharide (LPS) challenge, they also showed increased expression of a gene associated with maintaining gut integrity. Overall, this study shows that postbiotics from *V. proteolyticus* DCF12.2 can support intestinal health and help regulate immune responses in farmed fish.

## 1. Introduction

Fish serve as a vital component of the human diet, providing high-quality protein, essential fatty acids, and minerals necessary for growth and overall health [1,2]. In the context of aquaculture, it is important to evaluate various zootechnical, biological, and biochemical parameters, including growth performance [3]. Beyond these traditional metrics, recent advances have shifted attention toward nutritional strategies that not only support growth but also bolster fish health and farm sustainability. In this regard, functional feed additives have emerged as an innovative strategy to enhance sustainability, disease resistance, and resource optimization in aquaculture [4]. Among these additives, probiotics, which are live microorganisms that improve growth and confer health benefits to the host [5,6], have been widely included in aquafeeds [7,8].

Our research group previously identified *Vibrio proteolyticus* DCF12.2, isolated from healthy wedge sole (*Dicologlossa cuneata*), as a promising probiotic candidate due to its ability to enhance the immune response in fish, including the induction of cross-reactive antibody responses against fish pathogens [9,10]. This strain also exhibited other beneficial attributes, such as pathogen inhibition, non-virulence towards fish, resilience under storage conditions, and diverse hydrolytic activities (lecithinase, gelatinase, caseinase, amylase, and lipase), which could contribute to improving nutrient absorption in fish [9]. Moreover, it remained viable after passing through the fish gastrointestinal tract [10] and demonstrated protective effects against experimental infections with *Photobacterium damselae* subsp. *piscicida* and *Vibrio harveyi* [10], reinforcing its potential as a preventive strategy against fish diseases after dietary administration.

Nevertheless, the use of live probiotics poses certain risks, including horizontal gene transfer and the potential spread of antibiotic resistance, which may compromise their safety and efficacy [11]. To address these issues, the use of postbiotics has emerged as a promising alternative. In this sense, the International Scientific Association for Probiotics and Prebiotics (ISAPP) convened a panel that defined postbiotics as a “preparation of inanimate microorganisms and/or their components that confers a health benefit to the host” [12].

Postbiotic production traditionally relies on synthetic culture media formulated from refined substrates, which drives up costs and compromises sustainability, while energy-intensive processes further hinder large-scale use in aquafeeds [13]. Conversely, bacterial culture parameters—such as nutrient source, pH, and oxygen availability—can markedly influence the composition, stability, and bioactivity of the resulting postbiotic preparations [14]. Thus, in order to overcome these economic and environmental barriers, optimizing production using cost-effective agro-industrial by-products as culture substrates is critical for enhancing sustainability, efficacy, stability, and scalability of postbiotic production [15].

For instance, dietary supplementation with a cell-free extract derived from *Lactobacillus plantarum* significantly improved growth performance and stress resistance in white shrimp (*Penaeus vannamei*) [16]. Similarly, postbiotics derived from *Bacillus pumilus* have been shown to exert beneficial effects on the intestinal microbiota of grouper (*Epinephelus coioides*) [17] and digestive enzyme activity in gilthead seabream (*Sparus aurata*) [18], highlighting their potential for gut health improvement. These findings support the use of postbiotics as a promising strategy in aquafeeds, offering benefits in growth, immunity, and disease resistance when properly integrated [19].

Despite the well-characterized probiotic properties of *V. proteolyticus* DCF12.2, its potential as a postbiotic remains largely unexplored. Preliminary evaluations of the extracellular products (ECPs) produced by this strain under several culture conditions have revealed promising in vitro bioactivities, including stimulation of cellular proliferation, antibacterial and antibiofilm effects against fish pathogens, and enzymatic hydrolysis of both nutritional and antinutritional compounds. These effects were particularly evident when ECPs were obtained from *V. proteolyticus* DCF12.2 cultured in aquafeed-based media at 15 °C for 48 h. However, the in vivo effects of these postbiotic preparations within the fish gastrointestinal tract—a key organ for nutrient absorption and immune defense—remain unknown [20].

To address this gap, the present study evaluates the influence of dietary administration of a postbiotic obtained from *V. proteolyticus* DCF12.2 on gilthead seabream juveniles, focusing on its effects on intestinal histology, intestinal microbiota, and gene expression. Furthermore, at the end of the feeding trial, a lipopolysaccharide (LPS) challenge was conducted to analyze if the dietary administration of ECPs enhanced the fish’s immune response.

## 2. Materials and Methods

### 2.1. Bacterial Strain and Extracellular Product (ECPs) Extraction

*V. proteolyticus* DCF12.2 [9], originally isolated from healthy wedge sole (*D. cuneata*), was first cultured on tryptic soy agar (TSA; Oxoid Ltd., Basingstoke, UK) supplemented with 1.5% NaCl. After incubation at 23 °C for 24 h, one or two colonies were transferred to 50 mL of tryptic soy broth (TSB; Oxoid Ltd., Basingstoke, UK) with 1.5% NaCl and incubated at 23 °C for 12 h under shaking conditions (80 rpm) to reach the early stationary phase (approx. 10^9^ CFU mL^−1^).

ECPs were obtained by culturing the strain on a medium containing experimental aquafeed (160 g L^−1^) and agar (1.5%), following the method described by Liu [21] with modifications [14]. The aquafeed, provided by LifeBioencapsulation S.L., was previously characterized in detail [14]. Briefly, it was composed of the following ingredients (g/100 g): fishmeal, (10), soybean protein concentrate (15), wheat gluten (17), pea protein concentrate (5), soybean meal (20), wheat meal (14.14), fish oil (7), soybean oil (4.5), rapeseed oil (4.5), vitamins and minerals (1), vitamin C (0.05), vitamin E (0.5), and monocalcium phosphate (1.3).

After incubation, bacterial cells were harvested using 2 mL of sterile phosphate-buffered saline (PBS, pH 7.2), centrifuged (10,000× *g*, 20 min, 4 °C), and the resulting supernatants were sequentially filtered through 0.45 µm and 0.2 µm membrane filters (Merck Millipore, Billerica, MA, USA) to obtain cell-free ECPs. Protein concentration was quantified using the Qubit™ Protein Assay Kit and Qubit 2.0 fluorometer (Thermo Fisher Scientific, Waltham, MA, USA). ECPs were aliquoted and stored at −80 °C until use.

### 2.2. Experimental Diets

Two experimental diets were elaborated and produced at the Ceimar-Universidad de Almería facilities (Servicio de Piensos Experimentales): (i) a control diet (CTRL diet) mimicking the ingredient composition of commercial diets for gilthead seabream, including 20% fishmeal and 9.7% fish oil; (ii) a diet fortified with the ECPs solution (10 mL kg^−1^, ECP protein concentration 900 µg mL^−1^) applied to the feed pellets after cold-extrusion by using a vacuum fat coater (VP diet). The ingredients were first homogenized in a 10 L mixer, then finely ground with a hammer mill (UPZ 100, Hosokawa-Alpine, Augsburg, Germany) to 0.5 mm. The diets were cold-extruded in a two-screw extruder (Evolum 25, Clextral, France), fitted with 2 or 3 mm die holes. The extruder barrel consisted of four sections, and the temperature profile in each segment (from inlet to outlet) was 40, 40, 45, and 45 °C, respectively. Pellets were dried at 27 °C in a drying chamber (Airfrio, Almería, Spain) and cooled to room temperature. The ECP solution was applied to the diets the next day using a Pegasus PG-10VC LAB vacuum coater (Dinnissen, Sevenum, The Netherlands). Ingredient composition and proximate analysis of the diets are shown in Table 1. Proximate analysis of feeds was determined according to AOAC [22] procedures for dry matter and ash. Crude protein (N × 6.25) was determined by elemental analysis using a Fisons EA 1108 analyzer (Fisons Instruments, Beverly, MA, USA). Total lipid content was quantified following the procedure described by Folch et al. [23] using chloroform/methanol (2:1 *v*/*v*) as solvent.

### 2.3. Feeding Trial

Juvenile gilthead seabream (*S. aurata*) (32.7 ± 5.2 g) were obtained from a commercial hatchery (CUPIBAR, Chiclana de la Frontera, Cádiz, Spain) and acclimated to the experimental facilities at the Servicios Centrales de Investigación en Cultivos Marinos (SCI-CM, CASEM, University of Cádiz, Puerto Real, Cádiz; Spanish Operational Code REGA ES11028000312). Fish were maintained for 2 weeks in an open-flow seawater system under controlled conditions: temperature (19 °C), salinity (37 ppt), and natural photoperiod from January to March 2024 (36°31′45″ N, 6°11′31″ W). After acclimation, fish were randomly distributed into six 400 L tanks (n = 30 fish/tank; initial density 4.00 ± 0.02 kg m^−3^) and fed one of two experimental diets for 62 days: a control diet (CTRL diet) or a diet supplemented with ECPs from *V. proteolyticus* DCF12.2 (VP diet), each in triplicate. Fish were fed six times a week manually to apparent satiety twice daily. Diet identity was blinded to the personnel performing the feeding; feeds were labeled using color codes to eliminate observer bias.

All experimental procedures were performed in accordance with European Directive 2010/63/EU and Spanish legislation (RD 53/2013) regarding animal experimentation. Approval was granted by the Ethics and Animal Welfare Committee of the University of Cádiz and the Andalusian Regional Government (Junta de Andalucía, reference number 3/11/21/173).

### 2.4. Immunological Challenge

At the end of the feeding trial, six fish per tank (n = 18 per group) were randomly selected for a lipopolysaccharide (LPS) challenge. Prior to handling, fish were anesthetized with 2-phenoxyethanol (0.3 mL L^−1^). Fish from the CTRL group were intraperitoneally injected with either 0.1 mL of sterile saline (n = 3 per tank, n = 9 per group) or 0.1 mL of LPS (50 μg mL^−1^, Sigma-Aldrich, Madrid, Spain, #L4005) (n = 3 per tank, n = 9 per dietary treatment). The same procedure was applied to fish from the VP group (n = 3 per tank, n = 9 per treatment).

### 2.5. Fish Sampling

At the end of the 62-day feeding period, five fish per tank (n = 15 per dietary treatment) were randomly selected, fasted for 24 h, and euthanized with an overdose of 2-phenoxyethanol (1 mL L^−1^). Immediately after dissection, the abdominal cavity was opened, and the entire intestine was carefully removed. Whole intestines were preserved in DNA/RNA Shield (ZYMO Research) and stored at −80 °C for subsequent gene expression and intestinal microbiota analyses. Additionally, 1 cm sections of the proximal intestine from three fish per tank (n = 9 per dietary group) were excised and fixed for histological evaluation (see Section 2.6 and Section 2.7).

Following the intraperitoneal injection with saline or LPS solution, all challenged fish were sampled 72 h post-inoculation. The whole intestine was collected and stored at −80 °C for gene expression analysis.

### 2.6. Intestine Histology

Intestinal samples were fixed for 24 h in phosphate-buffered formalin (4% *v*/*v*, pH 7.2) at room temperature, and then dehydrated and embedded in paraffin following standard histological procedures. Transverse sections (5 μm) were cut to encompass the intestinal lumen. Slides were stained using hematoxylin and eosin (H&E) and examined under an Olympus IX51 light microscope equipped with a CC12 digital camera (Olympus Soft Imaging Solutions GmbH, Münster, Germany). Morphometric analysis was performed using ImageJ software (version 1.45; National Institutes of Health Image software, Bethesda, MD, USA). For each sample (9 fish per diet), 10 measurements per fish were recorded, assessing mucosal fold length, enterocyte height, *lamina propria* thickness, and goblet cell density (number per 100 μm of mucosal fold). These parameters were selected due to their sensitivity to dietary changes, particularly plant-derived ingredients [24].

### 2.7. Ultrastructural Study of the Intestinal Mucosa

At the end of the feeding trial, intestinal samples were collected for scanning (SEM) electron microscopy analysis. Tissues were fixed in 4% formaldehyde in phosphate buffer (pH 7.2) for 24 h at room temperature. They were then rinsed and passed through an ethanol gradient for dehydration. Samples were dried at the critical point using ethanol as the intermediate fluid and CO_2_ as the transition fluid (critical point dryer CDP 030, Leica Microsystems, Madrid, Spain). Dried samples were mounted on aluminum stubs, secured with colloidal graphite (PELCO Colloidal Graphite, Ted Pella Inc., Redding, CA, USA), and coated with gold using an SCD 005 Sputter Coater (Leica Microsystems, Madrid, Spain). SEM observations were conducted using a HITACHI S-3500 scanning electron microscope (Hitachi High Technologies Corporation, Tokyo, Japan). Digital images were processed with UTHSCSA ImageTool (University of Texas Health Sciences Center, San Antonio, TX, USA). SEM image data were used to estimate the apical area of enterocytes (EA) according to Vizcaíno et al. [25].

### 2.8. Microbiota Analysis from the Fish Gut

DNA was extracted from intestinal samples (n = 12 per dietary group) using a saline precipitation protocol [26], with modifications described by Tapia-Paniagua et al. [27]. A blank control using ddH_2_O was included to monitor contamination. DNA concentration was measured fluorometrically with the Qubit™ dsDNA HS Assay Kit (Thermo Fisher Scientific, Waltham, MA, USA), while purity and integrity were assessed using a NanoDrop™ One UV–Vis Spectrophotometer (Thermo Scientific, Wilmington, DE, USA) and 1% agarose gel electrophoresis, respectively.

The V3–V4 region of the 16S rRNA gene was amplified using the primers 5′-CCTACGGGNGGCWGCAG-3′ and 5′-GACTACHVGGGTATCTAATCC-3′ [28] and sequenced on an Illumina MiSeq platform (2 × 300 bp paired-end reads) at the Ultrasequencing Service of Novogene Europe (Munich, Germany).

Raw reads were quality-checked using FastQC (v0.11.9) [29]. Data processing, including trimming, error correction, and taxonomic assignment, was performed using the DADA2 pipeline with the SILVA v138 database [30], using a 99% similarity cutoff. Downstream microbiota analyses were conducted using the phyloseq and vegan packages in R [31,32]. Alpha diversity was assessed by calculating observed richness, Shannon, and Simpson diversity indices. Beta diversity was evaluated using non-metric multidimensional scaling (NMDS) based on Bray–Curtis dissimilarities. Amplicon sequence variants (ASVs) with fewer than 10 reads in at least 10% of the samples were removed. Taxonomic classification was reported at the phylum and genus levels.

Functional predictions of the microbial community were inferred using PICRUSt2 (v2.5) based on 16S rRNA gene data (https://github.com/picrust/picrust2/wiki, accessed on 20 October 2024).

### 2.9. Gene Expression Evaluation

Total RNA was extracted from the intestinal tissues of six fish per experimental group from the feeding trial using the GeneJET RNA Purification Kit (#K0732, Thermo Scientific), following the manufacturer’s protocol. The same procedure was applied to intestinal samples from fish injected with saline solution or LPS (n = 6 per group). RNA concentration was measured at 260 nm using a NanoDrop ND-1000 spectrophotometer, and RNA integrity was confirmed by agarose gel electrophoresis. Samples were stored at −80 °C until further use. To remove genomic DNA contamination, total RNA was treated with DNase I (Roche Diagnostics GmbH, Mannheim, Germany) following the manufacturer’s instructions. Reverse transcription was performed using the qScript cDNA Kit (Quanta BioSciences, Gaithersburg, MD, USA) with 1 μg of total RNA, and the resulting cDNA was stored at −20 °C.

Quantitative PCR (qPCR) was used to evaluate the relative expression of genes related to intestinal permeability and integrity—*cadherin 1* (*cdh1*), *cadherin 17* (*cdh17*), *integrin β6* (*itgb6*), *occludin* (*ocln*), and *zonula occludens-1* (*tjp1*)—as well as pro-inflammatory markers *tumor necrosis factor α* (*tnfα*) and *cyclooxygenase-2* (*cox2*) (Table 2). Expression levels were normalized using two reference genes: *elongation factor 1α* (*ef1α*) and *glyceraldehyde 3-phosphate dehydrogenase* (*gapdh*) (Table 2).

qPCR reactions were conducted in triplicate using a C1000 Touch™ thermal cycler with a CFX96™ optical module (Bio-Rad Laboratories, Madrid, Spain). Each reaction (10 μL final volume) contained 5 μL of GoTaq^®^ qPCR Master Mix (Promega Co., Madison, WI, USA), 0.5 μL each of forward and reverse primers (10 μM), 1 μL of cDNA, and 3 μL of nuclease-free water. qPCR cycling conditions followed the protocol described by Cerezo-Ortega et al. [36]. Amplification threshold cycle (Cq) values above 40 were considered negative. Relative mRNA expression was calculated using the 2^−ΔΔCq^ method [37], with normalization based on the geometric mean of the two reference genes and expression levels relative to the corresponding control group.

### 2.10. Statistical Analysis

Statistical differences in microbiota alpha diversity were determined using Student’s *t*-test, while differences in beta diversity between treatments were assessed via PERMANOVA. Predicted metabolic pathways were analyzed using the ALDEx2 tool following PICRUSt2 recommendations. Significantly different pathways were identified based on ALDEx2 “effect” size cutoffs of 0.5.

Histological parameters, taxonomic composition, and gene expression levels following the feeding trial were compared between experimental groups using Student’s *t*-test. For the experimental challenge, differences between groups were analyzed using two-way ANOVA followed by Tukey’s post hoc test. All data are expressed as mean ± standard deviation (SD), and differences were considered statistically significant at *p* ≤ 0.05.

All statistical analyses were performed using GraphPad Prism 9 (version 9.3.0; GraphPad Software, La Jolla, CA, USA).

## 3. Results

No fish mortality was recorded during the experimental period. Although growth performance was not a primary objective of this study, final body weight did not differ significantly between groups (70.1 ± 1.6 g in the CTRL group and 69.2 ± 2.1 g in the VP group; *p* > 0.05).

### 3.1. Effect of ECPs on Intestinal Histology and Ultrastructure

**Figure 1:**
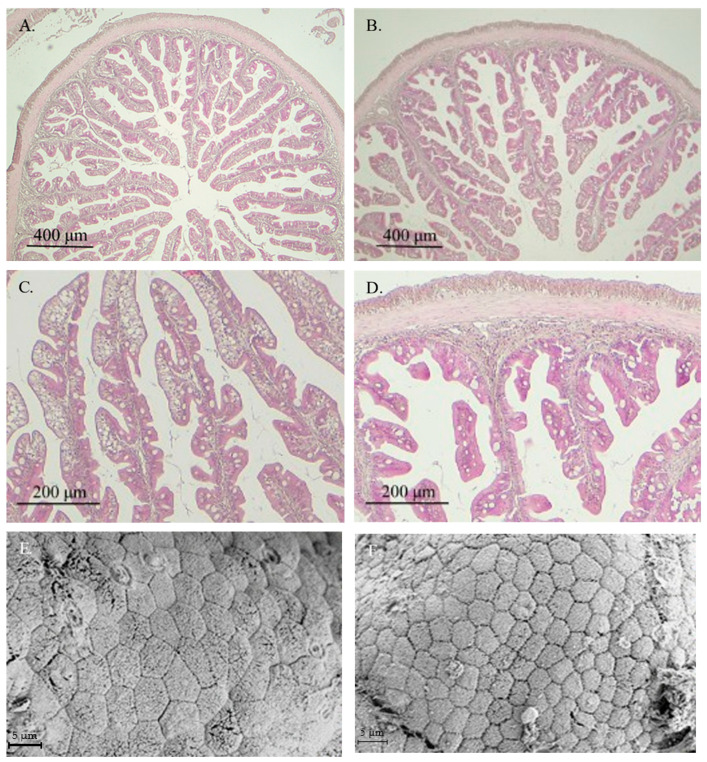
Images from light microscopy (**A**–**D**) and scanning electron microscopy (**E**,**F**) of the anterior intestinal region of gilthead seabream juveniles fed with CTRL (**A**,**C**,**E**) or VP (**B**,**D**,**F**) diets. No significant differences were observed between fish receiving both aquafeeds. CTRL: control diet; VP: ECPs of *V. proteolyticus* diet.

In general, healthy intestinal mucosa was observed in fish from both experimental groups, with no histological alterations or signs of enteritis in specimens receiving the VP diet (Figure 1B–D). Ultrastructural analysis showed similar healthy intestinal mucosa in fish fed both experimental diets (Figure 1E,F).

The histomorphometric data derived from those images are presented in Table 3. Fish fed the VP diet exhibited significantly shorter intestinal folds, reduced enterocyte height, and decreased thickness of the *lamina propria*, muscular layer, and submucosa compared to those fed the CT diet. In contrast, the number of goblet cells enhanced significantly in the VP group. Additionally, the apical area of the enterocytes was significantly larger in fish from the CT group than in those fed the VP diet.

### 3.2. Effect of ECPs on Intestinal Microbiota

No significant differences were observed in the number of observed ASVs between dietary groups (Table 4). Furthermore, Shannon and Simpson’s indices were found to be significantly higher in fish fed the VP diet.

**Figure 2:**
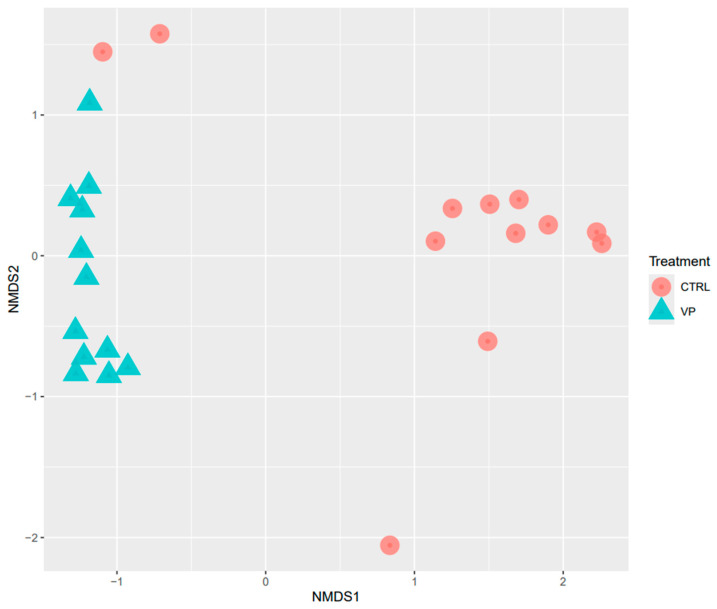
NMDS analysis of the intestine of gilthead seabream juveniles fed with the experimental diets. Codes are: CTRL: control diet (red circle); VP: ECPs of *V. proteolyticus* diet (blue triangle).

Beta diversity was analyzed using NMDS based on Bray–Curtis distances (Figure 2). The NMDS plot revealed statistically different clustering of microbial communities according to the dietary treatments (PERMANOVA, *p* = 0.001).

**Figure 3:**
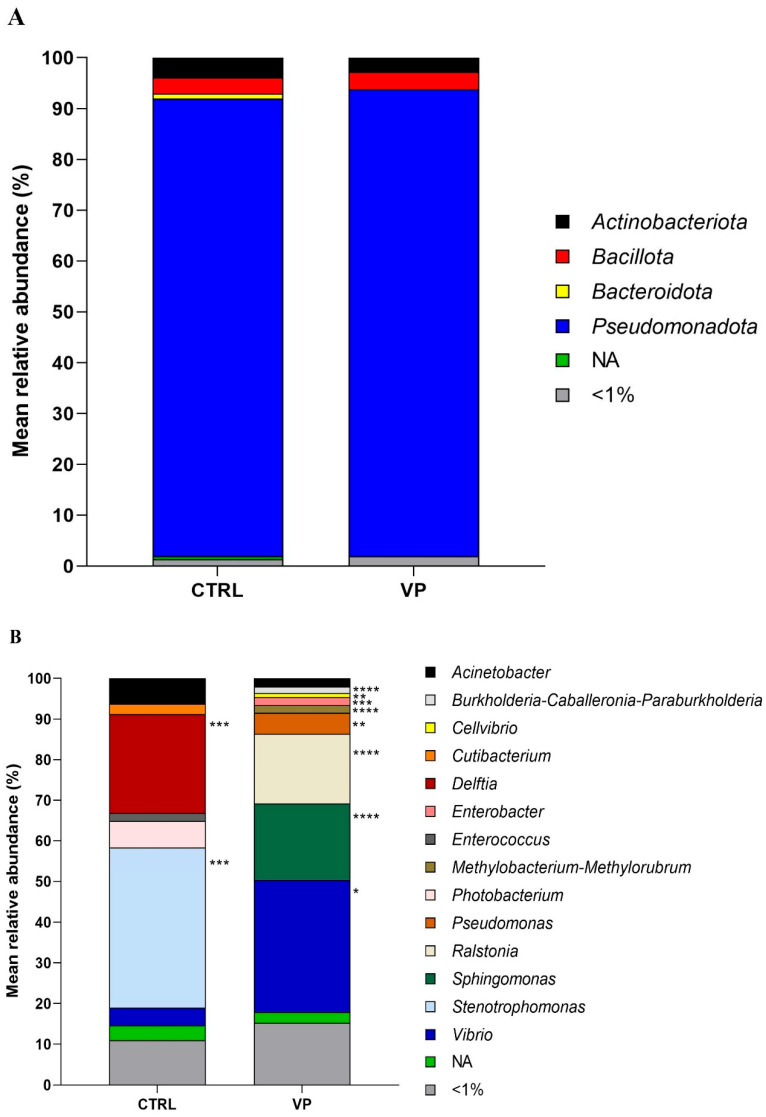
Relative abundance (%) of the taxa at the phylum (**A**) and genus (**B**) taxonomical categories in the intestine of gilthead seabream juveniles fed with the experimental diets. Codes are CTRL: control diet; VP: ECPs of *V. proteolyticus* diet. NA: Not assigned. Asterisks indicate significant differences between experimental groups (* *p* < 0.05; ** *p* < 0.01; *** *p* < 0.001; **** *p* < 0.0001).

The relative abundance of the most prevalent gut microbes at the phylum and genus levels is shown in Figure 3. The predominant phylum detected in fish from both dietary groups was *Pseudomonadota*, followed by *Actinobacteriota* and *Bacillota*. Although their relative abundances varied slightly depending on the diet, no significant differences were observed (Figure 3A).

At the genus level (Figure 3B), *Delftia*, *Enterococcus*, and *Stenotrophomonas* were abundant in the CTRL group but were completely absent in the VP group. In contrast, *Burkholderia-Caballeronia-Paraburkholderia*, *Cellvibrio*, and *Methylobacterium-Methylorubrum* were exclusively detected in the VP group. Among the shared genera, *Pseudomonas*, *Ralstonia*, *Sphingomonas*, and *Vibrio* showed significantly higher relative abundances in the VP group compared to the CTRL group. Additionally, *Acinetobacter*, *Cutibacterium*, and *Photobacterium* exhibited a slight, although not statistically significant, reduction in fish fed the VP diet.

Furthermore, predicted metabolic functionality did not show significant differences between the two dietary groups (Figure S1).

### 3.3. Influence of ECPs on Intestinal Gene Expression

**Figure 4:**
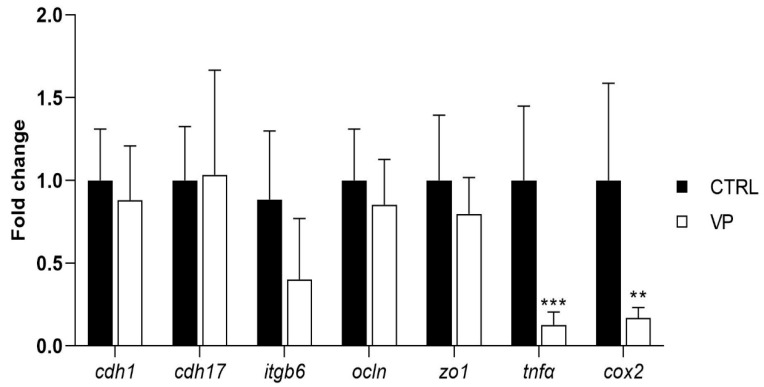
Relative quantification of selected gene expression in the intestine of gilthead seabream juveniles fed with the experimental diets. Data were normalized with *ef1α* and *gadph* transcription levels and expressed as mean ± SD (n = 6) of fold change. Codes are CTRL: control diet; VP: ECPs of *V. proteolyticus* diet. Student’s *t*-test was used. Asterisks indicate significant differences between experimental groups (** *p* < 0.01; *** *p* < 0.001).

The expression levels of genes related to intestinal permeability and integrity (*cdh1, cdh17, itgb6, ocln*, and *zo1*) did not show significant differences between dietary treatments (Figure 4). However, fish fed the VP diet exhibited a downregulation of the pro-inflammatory markers *tnfα* and *cox2* compared to the CTRL group (Figure 4).

### 3.4. Effect of LPS Challenge on Intestinal Gene Expression

**Figure 5:**
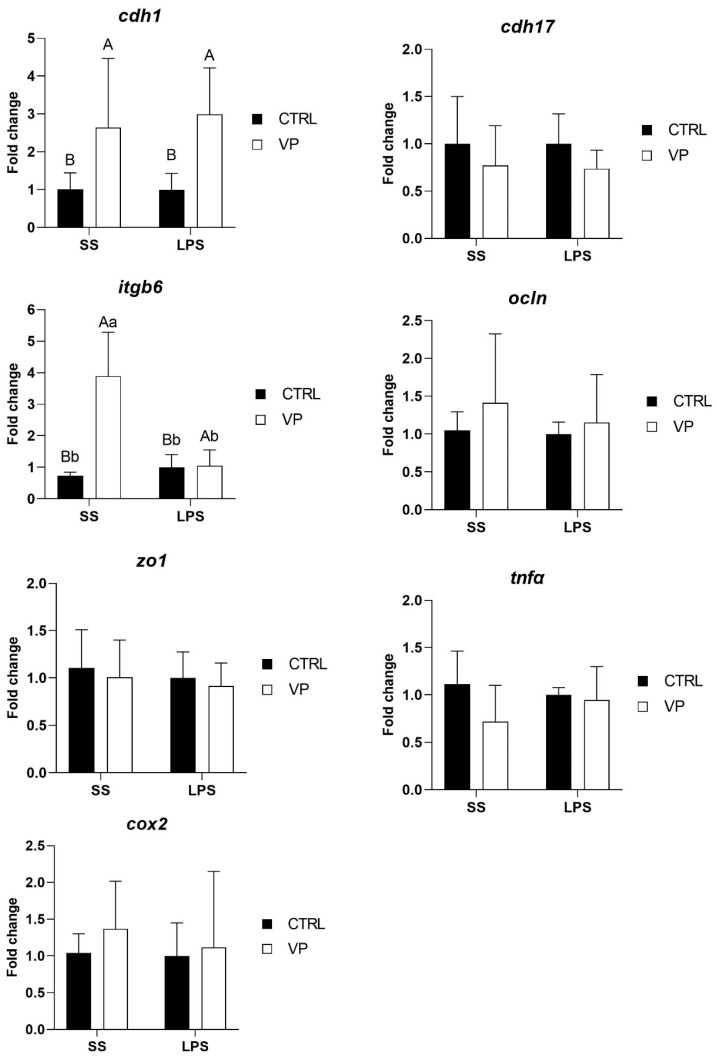
Relative quantification of selected gene expression in the intestine of gilthead seabream juveniles fed with the experimental diets and subjected to experimental challenge. Data were normalized with *ef1α* and *gadph* transcription levels and expressed as mean ± SD (n = 6) of fold change. Codes are CTRL: control diet; VP: ECPs of *V. proteolyticus* diet; SS: challenge with Saline solution; LPS: challenge with LPS. Differences between groups were analyzed using two-way ANOVA, followed by Tukey’s post hoc test, and were considered statistically significant at *p* ≤ 0.05. Different uppercase letters indicate significant differences due to the diet, and lowercase letters indicate significant differences due to the LPS challenge.

Following the LPS challenge, *cdh1* transcription level was affected by the diet, being significantly higher in fish fed the VP diet, but not by the challenge (Figure 5, Table 5). Furthermore, the *itgb6* expression level was upregulated significantly in fish from the VP group injected with saline solution. No significant differences were observed in the transcription levels of *cdh17, ocln, zo1*, *tnfα*, and *cox2* between dietary groups following the challenge.

## 4. Discussion

The use of postbiotics—non-viable microbial cells or their metabolic byproducts that confer health benefits to the host—has gained growing interest in aquaculture as a safe and effective alternative to traditional probiotics. In the present study, we evaluated the effects of dietary supplementation with extracellular products (ECPs) derived from *Vibrio proteolyticus* grown on a medium containing experimental aquafeed and agar, focusing on their impact on intestinal histology, microbiota composition, and gene expression in juvenile *Sparus aurata*. This specific culture condition was selected due to its ability to produce *V. proteolyticus* ECPs that promote cellular proliferation, have antibacterial and antibiofilm activity against fish pathogens, and display a variety of enzymes to hydrolyse nutritional and antinutritional compounds. Although growth performance was not the primary objective, both experimental diets were well tolerated, and no significant differences in final body weight were observed between groups.

Histological assessment revealed that fish fed the VP diet exhibited a well-preserved mucosal structure in the anterior intestinal region, with no apparent signs of enteritis or tissue damage, suggesting the absence of any deleterious effects associated with postbiotic administration. However, a significant reduction in several mucosal parameters, including fold height, enterocyte height, and the thickness of the *lamina propria*, muscular layer, and submucosa, was observed in the VP group compared to the control. These reductions might be interpreted as a potential compromise in the absorptive surface area [38,39]. Nonetheless, some authors have described such morphological changes as regulatory adjustments in response to functional aquafeeds or microbial-derived compounds, rather than pathological alterations (see review by De Marco et al. [40]).

In this context, the VP diet significantly enhanced the number of goblet cells in the intestinal mucosa. Goblet cells play a central role in maintaining mucosal integrity by secreting mucins that form the protective mucus layer over the epithelium [41]. An increase in goblet cell density is often considered a marker of enhanced mucosal protection and improved epithelial defense, especially under microbial stimulation or in response to immunomodulatory interventions [42]. Additionally, the reduced apical area of enterocytes observed in the VP group may reflect subtle alterations in membrane dynamics or nutrient absorption potential. However, since no differences were observed in fish growth performance, these histological changes do not appear to negatively affect nutrient assimilation and may instead represent a shift toward a more compact and regulated epithelial profile.

The dietary fortification with ECPs significantly influenced the intestinal microbial diversity and composition. Alpha diversity, as reflected by the Shannon and Simpson indices, was significantly higher in the VP group. This observation is consistent with previous studies reporting enhanced alpha diversity in fish fed postbiotic-supplemented diets [43,44]. High microbial diversity is generally associated with improved gut health, metabolic resilience, and enhanced resistance to pathogen colonization in fish [45,46]. According to this idea, the inclusion of ECPs in the aquafeed can be beneficial by improving the physiological state of the fish. Furthermore, beta diversity analysis revealed significant differences in microbial community between dietary groups, confirming the modulatory effect of the VP postbiotic diet on the intestinal microbiota of these individuals.

At the phylum level, the microbiota was dominated by *Pseudomonadota*, *Actinobacteriota*, and *Bacillota* in both groups, consistent with previous studies in gilthead seabream [36,47,48]. However, more detailed taxonomic analysis at the genus level revealed marked differences. In the control group, *Delftia*, *Enterococcus*, and *Stenotrophomonas* were abundant but completely absent in the VP group. Interestingly, some *Enterococcus* strains are known as opportunistic or pathogenic potential bacteria, and their reduction may be favorable in the context of fish health [49,50].

In contrast, the VP diet promoted the exclusive presence of *Burkholderia-Caballeronia-Paraburkholderia*, *Cellvibrio*, and *Methylobacterium-Methylorubrum*. Members of the *Burkholderia-Caballeronia-Paraburkholderia* group are known for their broad-spectrum antimicrobial activity [51] and their role in bioremediation due to their ability to degrade aromatic compounds [52]. *Cellvibrio* species are able to degrade complex polysaccharides such as cellulose, xylan, and pectin [53], while *Methylobacterium-Methylorubrum* has been associated with enhanced energy metabolism [54]. These shifts suggest a selective pressure exerted by the postbiotic, fostering microbial taxa with metabolic capacities adapted for complex substrate utilization or beneficial interactions with the intestinal mucosa.

Moreover, the significant enrichment of *Pseudomonas*, *Ralstonia*, *Sphingomonas*, and *Vibrio* in the VP group is notable. Although some *Vibrio* species are known to be pathogenic to fish [55,56], others (including *V. proteolyticus*) have been shown to exhibit probiotic-like properties or immune stimulation potential in fish [10,57]. *Pseudomonas* and *Sphingomonas* species are known to have a wide range of metabolic capabilities, produce antimicrobial compounds, mitigate intestinal inflammation, and modulate host immunity [58,59,60,61,62]. Likewise, *Ralstonia* has been associated with protein digestion and absorption, as well as pathways involved in phenylalanine metabolism, ketone body synthesis and degradation, and lysine catabolism [63]. Altogether, these findings suggest that the postbiotic may promote a more dynamic and potentially beneficial microbial community.

Importantly, despite these microbial shifts, predictive metabolic functionality analyses did not reveal significant differences between dietary treatments. This indicates that while taxonomic composition was altered, the overall functional capacity of the gut microbiota remained stable. This could be due to functional redundancy, where different microbial taxa perform similar metabolic roles [64,65]. For future studies, it would be interesting to check whether these changes in the microbial structure confer protection to specimens that have received aquafeed supplemented with ECPs and are subjected to different changes typical of aquaculture practice (hypoxia, fasting, temperature changes, etc.).

The expression levels of genes associated with intestinal integrity (*cdh1*, *cdh17*, *itgb6*, *ocln*, and *zo1*) did not differ significantly between dietary groups. These genes play crucial roles in maintaining epithelial cohesion, regulating paracellular permeability, and ensuring intestinal homeostasis [66,67]. It has been demonstrated in *S. aurata* that the alteration of such genes due to the presence of mycotoxins in the aquafeed induces a dysregulation of intestinal physiology [68]; however, this is not appreciated when including ECPs in the aquafeed. The absence of changes at the level of intestinal integrity biomarkers is in agreement with histological analysis, suggesting that the inclusion of *V. proteolyticus* extracellular products did not compromise the structural integrity of the intestinal barrier. The stability in their expression levels suggests that the VP diet did not induce gut barrier dysfunction, a key concern when evaluating new dietary additives.

Interestingly, fish fed the VP diet exhibited significant downregulation of *tnfα* and *cox2*, two key genes involved in inflammation. *tnfα* (tumor necrosis factor-alpha) is a pro-inflammatory cytokine that plays a central role in initiating immune responses and is commonly upregulated during intestinal stress or pathogenic challenges [69,70]. *cox2* (cyclooxygenase-2) is an enzyme responsible for the synthesis of prostaglandins, which mediate inflammatory responses in the gut [71]. The lower transcription levels of these genes in fish fed the VP diet suggest a reduction in basal inflammation status, which may indicate a beneficial immunomodulatory effect of the dietary treatment.

This reduction in transcription levels of key inflammatory genes may be due to specific changes in the intestinal microbiota. As mentioned above, *Stenotrophomonas* and *Delftia*, both detected in the CTRL group, were completely absent in the VP group. Both genera have been implicated in pro-inflammatory responses [72,73]. Their disappearance in fish fed the VP diet could indicate that the ECPs from *V. proteolyticus* may exert a selective pressure on the gut microbiota, limiting the presence of potentially pro-inflammatory bacteria. This microbial shift, in turn, could contribute to the observed downregulation of inflammatory markers, highlighting a possible microbiota-immune axis modulated by the dietary postbiotic.

Notably, the decrease in *tnfα* and *cox2* expression without alterations in tight junction or adhesion-related genes (see above) may imply that ECPs incorporated into the diet contribute to maintaining gut homeostasis by reducing unnecessary inflammatory signaling. Chronic intestinal inflammation can lead to tissue damage, increased permeability, and impaired nutrient absorption [74]. Therefore, the observed reduction in inflammation-related gene expression could reflect a more balanced immune status, which may be advantageous for long-term gut health and overall fish performance.

After the LPS challenge, the expression of *cdh1* was significantly higher in fish fed the VP diet, suggesting a potential dietary influence on epithelial integrity. *cdh1* encodes E-cadherin, a key adhesion molecule essential for maintaining epithelial structure and intestinal barrier function [66]. The upregulation of *cdh1* in fish fed the VP diet may indicate a protective effect of the dietary treatment, potentially enhancing epithelial resilience against stressors. Notably, this effect was attributed to the diet rather than the LPS challenge itself, suggesting a preconditioning effect of the VP diet in reinforcing intestinal epithelial stability.

Interestingly, *itgb6* expression showed a complex pattern of change. Before the challenge, *itgb6* expression levels were lower in VP-fed fish compared to the CTRL group, although this reduction was not statistically significant (Figure 4). However, after the challenge, *itgb6* was significantly upregulated in VP-fed fish injected with saline but not in those challenged with LPS (Figure 5). *itgb6* encodes integrin β6, which plays a key role in epithelial repair and immune regulation, particularly in response to tissue injury [75]. The initial non-significant reduction in *itgb6* before the challenge may suggest a lower basal need for epithelial remodelling in VP-fed fish, possibly reflecting a more stable intestinal environment. The significant post-injection upregulation of *itgb6* in response to saline, but not to LPS, may indicate that the VP diet modulated the epithelial response to mild perturbations while preventing excessive activation during inflammatory stimulation.

Furthermore, no significant differences were observed in the transcription levels of *cdh17*, *ocln*, *zo1*, *tnfα*, and *cox2* between fish fed the CTRL and VP diets after the challenge (Figure 5). The stability in tight junction (*ocln, zo1*) and cadherin (*cdh17*) expression suggests that the VP diet did not compromise gut barrier function under inflammatory conditions. Moreover, the absence of differences in *tnfα* and *cox2* expression post-challenge indicates that the VP diet did not exacerbate or suppress the acute inflammatory response triggered by LPS. Given that these genes were downregulated before the challenge (Figure 4), this suggests that the VP diet may have conferred a baseline anti-inflammatory effect, rather than altering the immediate immune response to LPS exposure.

## 5. Conclusions

In conclusion, the dietary inclusion of a postbiotic derived from *V. proteolyticus* DCF12.2 modulated the intestinal status of *S. aurata* specimens by increasing goblet cell numbers, promoting microbial diversity, reducing inflammatory gene expression, and enhancing epithelial gene responses under immune challenge. These findings highlight the immunomodulatory and gut-health-promoting potential of postbiotics as promising, stable alternatives to probiotics in aquafeeds. Further research should aim to validate their efficacy under commercial farming conditions and in response to pathogenic exposure, while optimizing postbiotic production processes for large-scale application.

## Figures and Tables

**Table 1:** Ingredient and chemical composition (% dry basis) of the experimental diets used in the feeding trial.

Ingredient (% Dry Basis)	CTRL	VP
Fishmeal LT94 ^1^	20.0	20.0
Lysine ^2^	1.2	1.2
Methionine ^3^	0.5	0.5
Squid meal ^4^	2.0	2.0
CPSP90 ^5^	1.0	1.0
Krill meal ^6^	2.0	2.0
Wheat gluten ^7^	10.0	10.0
Soybean protein concentrate ^8^	8.5	8.5
Soybean meal ^9^	8.5	8.5
Pea protein concentrate ^10^	6.0	6.0
Fish oil ^11^	9.7	9.7
Vegetable oil ^12^	2.0	2.0
Soybean lecithin ^13^	1.0	1.0
Wheat meal ^14^	25.0	25.0
Monocalcium phosphate ^15^	0.5	0.5
Vitamin and Mineral premix ^16^	2.0	2.0
Vitamin C ^17^	0.1	0.1
ECPs from *V. proteolyticus* (mL) ^18^	0	1
Crude protein	48.5	49.5
Crude lipid	17.5	17.1
Ash	7.0	7.6
Moisture	5.8	5.7

Dietary codes: CTRL: control diet without ECPs; VP: diet fortified with *V. proteolyticus* ECPs; ^1^ 69.4% crude protein, 12.3% crude lipid (Norsildemel, Bergen, Norway); ^2, 3^ Lorca Nutrición Animal SA (Murcia, Spain); ^4, 5, 6^ purchased from Bacarel (UK). CPSP90 is enzymatically pre-digested fishmeal; ^7^ 78% crude protein (Lorca Nutrición Animal SA, Murcia, Spain). ^8^ Soycomil, 60% crude protein, 1.5% crude lipid (ADM, Poland); ^9^ Lorca Nutrición Animal SA (Murcia, Spain); ^10^ pea protein concentrate, 85% crude protein, 1.5% crude lipid (Emilio Peña SA, Spain); ^11^ AF117DHA (Afamsa, Spain); ^12^ blend of soybean, rapeseed and linseed (4:4:2) oils (Aceites el Niño, Spain); ^13^ P700IP (Lecico, DE); ^14^ local provider (Almería, Spain); ^15^ Lorca Nutrición Animal SA (Murcia, Spain); ^16^ *Lifebioencapsulation* SL (Almería, Spain). Vitamins (mg kg^−1^): vitamin A (retinyl acetate), 2,000,000 UI; vitamin D3 (DL-cholecalciferol), 200,000 UI; vitamin E (Lutavit E50), 10,000 mg; vitamin K3 (menadione sodium bisulphite), 2500 mg; vitamin B1(thiamine hydrochloride), 3000 mg; vitamin B2 (riboflavin), 3000 mg; calcium pantothenate, 10,000 mg; nicotinic acid, 20,000 mg; vitamin B6 (pyridoxine hydrochloride), 2000 mg; vitamin B9 (folic acid), 1500 mg; vitamin B12 (cyanocobalamin), 10 mg vitamin H (biotin), 300 mg; inositol, 50,000 mg; betaine (Betafin S1), 50,000 mg. Minerals (mg kg^−1^): Co (cobalt carbonate), 65 mg; Cu (cupric sulphate), 900 mg; Fe (iron sulphate), 600 mg; I (potassium iodide), 50 mg; Mn (manganese oxide), 960 mg; Se (sodium selenite), 1 mg; Zn (zinc sulphate) 750 mg; Ca (calcium carbonate), 18.6%; (186,000 mg); KCl, 2.41%; (24,100 mg); NaCl, 4.0% (40,000 mg); ^17^ TECNOVIT, Spain; ^18^ ECPs of *V. proteolyticus* DCF12.2.

**Table 2:** List of genes studied in this work.

Product Gene	Code	Reference
**Permeability and integrity**		
Cadherin 1	*cdh1*	Pérez-Sánchez et al. [33]
Cadherin 17	*cdh17*	Pérez-Sánchez et al. [33]
Integrin 6-β	*itgb6*	Pérez-Sánchez et al. [33]
Ocludin	*ocln*	Pérez-Sánchez et al. [33]
Zonula-occludens 1	*tjp1*	Cerezuela et al. [34]
**Pro-inflammatory**		
Tumor necrosis factor α	*tnf* *α*	Estruch et al. [35]
Cyclooxygenase 2	*cox2*	Estruch et al. [35]
**Reference genes**		
Elongation factor 1α	*ef1α*	Estruch et al. [35]
Ribosomal glyceraldehyde 3-phosphate dehydrogenase	*gadph*	Estruch et al. [35]

**Table 3:** Histomorphometric analysis of the intestinal mucosa in juvenile gilthead seabream fed with the experimental diets.

	CTRL	VP	*p*
FL (µm)	805.92 ± 124.96	508.61 ± 69.82 *	<0.0001
EH (µm)	37.67 ± 5.94	21.41 ± 3. 53 *	<0.0001
LP (µm)	25.71 ± 6.59	19.01 ± 4.66 *	<0.0001
ML (µm)	38.82 ± 10.01	25.15 ± 4.69 *	<0.0001
SBL (µm)	26.69 ± 9.84	19.74 ± 4.66 *	<0.0001
GC	6.99 ± 1.22	9.47 ± 1.67 *	<0.0001
AE	26.94 ± 3.25	14.97 ± 2.16 *	<0.0001

CTRL: control diet; VP: ECPs of *V. proteolyticus* diet; FL: fold length; EH: enterocyte height; LP: *Lamina propria* thickness; ML: muscular layer thickness; SBL: submucosa layer thickness; GC: number of goblet cells per µm; AE: enterocyte apical area. Data represents mean ± SD. Student’s *t*-test was used, and differences were considered statistically significant at *p* ≤ 0.05. The asterisks indicate statistically significant differences between treatments.

**Table 4:** Alpha diversity indices in the intestine of gilthead seabream juveniles fed with the experimental diets.

	CTRL	VP	*p*
**Observed**	330.30 ± 116.10	277.00 ± 93.14	0.2364
**Shannon**	2.14 ± 0.51	3.30 ± 0.70 *	0.0183
**Simpson**	0.64 ± 0.23	0.92 ± 0.02 *	0.0019

CTRL: control diet; VP: ECPs of *V. proteolyticus* diet; values are shown as the mean ± SD (n = 12 per diet). Student’s *t*-test was used, and differences were considered statistically significant at *p* ≤ 0.05. Asterisks indicate significant differences between experimental groups.

**Table 5:** Statistical parameters (*p*-value) obtained from two-way ANOVA analysis of fish fed the experimental diets subjected to the LPS challenge. Asterisks indicate significant differences (*p* < 0.05).

Two-Way ANOVA	Diet	Challenge	Interaction
*cdh1*	0.0009 *	0.7113	0.6878
*cdh17*	0.1247	0.9168	0.9347
*itgb6*	0.0001 *	0.0009 *	0.0002 *
*ocln*	0.2774	0.5258	0.6579
*zo1*	0.5172	0.4938	0.9581
*tnfα*	0.0993	0.6587	0.2032
*cox2*	0.4194	0.5972	0.7003

## Data Availability

The data that support the findings of this study are available from the corresponding author upon reasonable request.

## Supplementary Materials

The following supporting information can be downloaded at: https://www.mdpi.com/article/10.3390/ani15131982/s1, Figure S1: Metabolic functionality in the intestine of gilthead seabream juveniles fed with the experimental diets. Codes are: CTRL: control diet; VP: ECPs of *V. proteolyticus* diet.

## Abbreviations

The following abbreviations are used in this manuscript:

ISAPP	International Scientific Association for Probiotics and Prebiotics
UTHSCSA	University of Texas Health Sciences Center, San Antonio
SCI-CM	Servicios Centrales de Investigación en Cultivos Marinos
ECPs	Extracellular Product
LPS	Lipopolysaccharide
CRTL	Control
VP	*Vibrio proteolyticus*
qPCR	Quantitative PCR
TSA	Tryptic soy agar
TSB	Tryptic soy broth
CFU	Colony-Forming Unit
SEM	Scanning Electron Microscope
EA	Apical area of enterocytes
NMDS	Non-metric multidimensional scaling
ASVs	Amplicon sequence variants
Cq	Quantification Cycle
SD	Standard deviation
FL	Fold length
EH	Enterocyte height
LP	*Lamina propria*
ML	Muscular layer
SBL	Submucosa layer thickness
GC	Goblet cells
AE	Enterocyte apical area
NMDS plot	Non-metric Multidimensional Scaling plot
NA	Not assigned
