# Supplementary material for: Dietary Administration of Postbiotics from Vibrio proteolyticus DCF12.2 Enhanced Intestinal Integrity, Microbiota, and Immune Response in Juvenile Gilthead Seabream (Sparus aurata)
Source: Animals (Basel). 2025 Jul 5;15(13):1982. doi: 10.3390/ani15131982 (PMC12248857; doi:10.3390/ani15131982)
Supplement: Supplementary file 1 [file animals-15-01982-s001.zip › animals-3696399-supplementary.pdf]

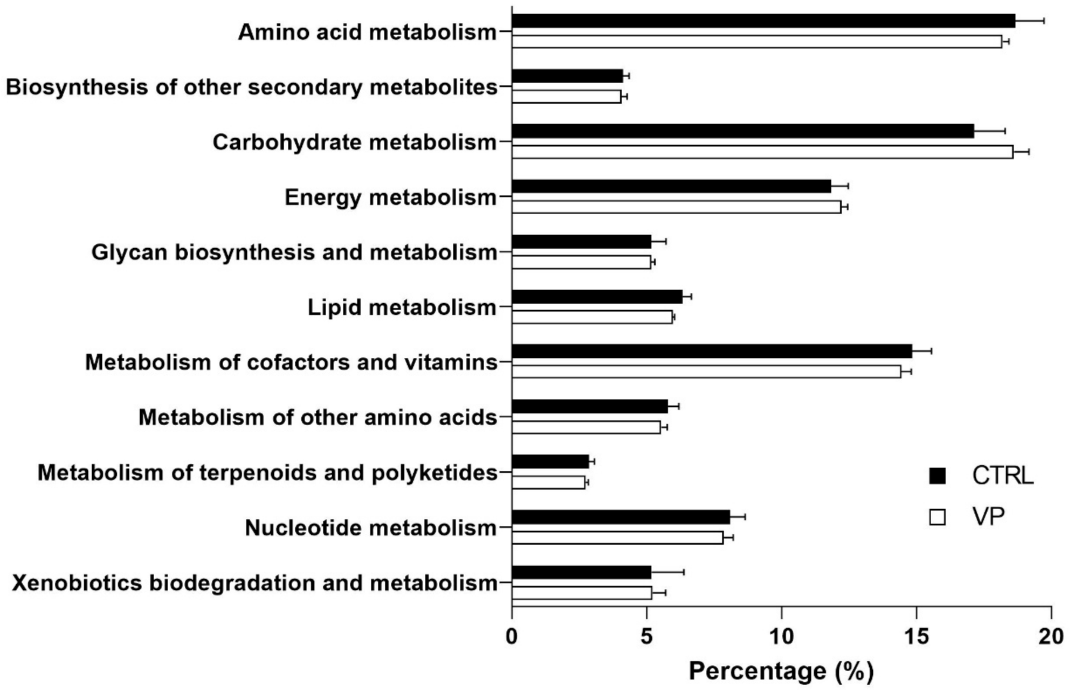

Figure S1. Metabolic functionality in the intestine of gilthead seabream juveniles fed with the experimental diets. Codes are: CTRL: control diet; VP: ECPs of *V. proteolyticus* diet.
